# HPLC–ICP-MS speciation analysis and risk assessment of arsenic in *Cordyceps sinensis*

**DOI:** 10.1186/s13020-018-0178-9

**Published:** 2018-04-16

**Authors:** Tian-Tian Zuo, Yao-Lei Li, Hong-Yu Jin, Fei Gao, Qi Wang, Ya-Dan Wang, Shuang-Cheng Ma

**Affiliations:** National Institutes for Food and Drug Control, No. 2 Tiantan Xili, Dongcheng District, Beijing, 100050 China

**Keywords:** Arsenic, Speciation, Inductively coupled plasma mass spectrometry (HPLC–ICP-MS), Risk assessment, Hazard index (HI), Lifetime cancer risk (CR), Target hazard quotient (THQ)

## Abstract

**Background:**

*Cordyceps sinensis*, one of the most valued traditional herbal medicines in China, contains high amount of arsenic. Considering the adverse health effects of arsenic, this is of particular concern. The aim of this study was to determine and analyze arsenic speciation in *C. sinensis*, and to measure the associated human health risks.

**Methods:**

We used microwave extraction and high-performance liquid chromatography coupled with inductively coupled plasma mass spectrometry to determine and analyze the arsenic content in *C. sinensis*, and measured the associated human health risks according to the hazard index (HI), lifetime cancer risk (CR), and target hazard quotient (THQ).

**Results:**

The main arsenic speciation in *C. sinensis* were not the four organic arsenic compounds, including dimethyl arsenic, monomethyl arsenic, arsenobetaine, and arsenocholine, but comprised inorganic arsenic and other unknown risk arsenic compounds. HI scores indicated that the risk of *C. sinensis* was acceptable. CR results suggested that the cancer risk was greater than the acceptable lifetime risk of 10^−5^, even at low exposure levels. THQ results indicated that at the exposure level < 2.0 months/year, the arsenic was not likely to harm human health during a lifetime; however, if the exposure rate was > 3.0 months/year, the systemic effects of the arsenic in *C. sinensis* was of great concern.

**Conclusion:**

The arsenic in *C. sinensis* might not be free of risks. The suggested *C. sinensis* consumption rate of 2.0 months/year provided important insights into the ways by which to minimize potential health risks. Our study not only played the role of “cast a brick to attract jade” by which to analyze arsenic speciation in *C. sinensis* but also offered a promising strategy of risk assessment for harmful residues in traditional herbal medicines.

**Electronic supplementary material:**

The online version of this article (10.1186/s13020-018-0178-9) contains supplementary material, which is available to authorized users.

## Background

*Cordyceps sinensis* (Berk.) Sacc. is the Chinese caterpillar fungus that consists of the stromata of the fungus. This fungus is also known as “winter worm summer grass” because of its appearance during different seasons [1]. *C. sinensis* is distributed mostly in Tibet and in the Qinghai, Sichuan, Yunnan, and Gansu Provinces in China at an altitude of 3500–5000 m. With various biological activities, *C. sinensis* has been considered as one of the most well-known and valued traditional medicinal herbs and has been used extensively for ailing patients in China and other Asian countries over the past 300 years. It is also officially listed in Pharmacopoeia of the People’s Republic of China (PPRC) [2]. Its proposed therapeutic benefits include antitumor, antioxidant, antidiabetic, hepatoprotective, nephroprotective, diuretic, anti-inflammatory, and soothing asthma effects [3–9].

Heavy metals are ubiquitous environmental pollutants resulting from natural or anthropogenic activities such as waste disposal, coal combustion, mining, pesticides application and chemical fertilizer use, which can lead to the contamination of traditional herbal medicines, including *C. sinensis*. Prolonged exposure to toxic heavy metals is responsible for a variety of detrimental effects on the health of the human body [10]. Recent studies have demonstrated the high content of heavy metals, specifically arsenic, in *C. sinensis* [11, 12].

Arsenic is a ubiquitous metalloid and is considered to be one of the most significant pollutants in the world, existing in both organic and inorganic forms. Different arsenic speciation displays different toxicity; therefore, it is important to identify this speciation in *C. sinensis*. It was reported that organic arsenic, found mainly as arsenobetaine (AsB), arsenosugars, and arsenolipids, presents often in seafood and is relatively nontoxic [13, 14]; however, inorganic arsenic, including As III and As V, has been demonstrated to be a human group I carcinogen without threshold [15]. Although the carcinogenic mechanisms of inorganic arsenic are not clear, it has been proposed that because of the interaction of inorganic arsenic with cysteine residues in zinc finger domains, the formation of complexes with inorganic arsenic results in the substitution of zinc and consequently DNA damage and the loss of protein function [16, 17]. Other health effects of inorganic arsenic include skin lesions, cardiovascular problems, diabetes, kidney dysfunction, respiratory infection, and immune system diseases [17–22]. For pregnant women, chronic inorganic arsenic exposure has caused excessive stillbirth, spontaneous abortion, and preterm birth rates [23]. In the previous study, the contents of high toxic elements including lead, cadmium, total mercury and total arsenic in *C. sinensis* were determined. The results displayed that except arsenic, the levels of other three types of heavy metals in *C. sinensis* were far below the limit for herbal medicines in PPRC [2]. However, the contents of total arsenic in *C. sinensis* were about four times higher than the arsenic limit for herbal medicines recorded in PPRC [2]. Considering the increase in the popularity of *C. sinensis* use in China in recent years and considering public health safety, it is crucial to determine the level of arsenic speciation in *C. sinensis* and the associated risks to human health. The objectives of our study are as follows: (1) determine the concentrations of total arsenic in *C. sinensis*; (2) develop method by which to determine the concentration levels of different speciation of arsenic in *C. sinensis* and compare them with total arsenic; (3) assess the associated potential risks to human health using hazard index (HI), cancer risk (CR), and target hazard quotient (THQ) scales by the rule of risk maximization and protecting most people, and provide consumption suggestions for *C. sinensis* to minimize these risks.

## Methods

The Minimum Standards of Reporting Checklist contains details of the experimental design, and statistics, and resources used in this study (Additional file 1).

### Reagents

Doubly deionized water was prepared using the Milli-Q (Millipore, Milford, MA, USA) water purification system. The arsenic mono-element standard solution (100.0 μg/mL), dimethyl arsenic (DMA), monomethyl arsenic (MMA), As III, As V, AsB, and arsenocholine (AsC) standard solution were purchased from National Standard Material Research Center (Beijing, China). The internal standard solution containing germanium (m/z = 74, 100.0 μg/mL) was purchased from Agilent (Agilent Technologies, Folsom, CA, USA). Suprapur trace metal grade concentrated nitric acid (HNO_3_, 65.0%) was purchased from Merck (Merck, Munchen, Germany). Analytical grade ammonium carbonate ([NH_4_]_2_CO_3_) was purchased from Beijing Chemical Reagent Co. (Beijing, China).

### Herbs

Thirty four samples of *C. sinensis* were collected in batches from traditional herbal-medicine markets or retail pharmacies in Tibet and in Qinghai, Yunnan, and Sichuan Provinces, the areas in which *C. sinensis* is the most highly distributed in China (Table 1). All samples were authenticated by Mr. Shuai Kang. The voucher specimens were deposited in National Institutes for Food and Drug Control (NIFDC), Beijing, China.

**Table 1:** Sample collection information in present study

Code	Collecting time	Location	Source
01	2016, 06	Yushu, Qinghai	Production site
02	2016, 06	Yushu, Qinghai	Production site
03	2016, 05	Guoluo, Qinghai	Production site
04	2016, 05	Guoluo, Qinghai	Production site
05	2016, 06	Guoluo, Qinghai	Production site
06	2016, 06	Guoluo, Qinghai	Production site
07	2016, 05	Hainan, Qinghai	Production site
08	2016, 06	Hainan, Qinghai	Production site
09	2016, 06	Hainan, Qinghai	Production site
10	2016, 05	Huangnan, Qinghai	Production site
11	2016, 05	Huangnan, Qinghai	Production site
12	2016, 06	Huangnan, Qinghai	Production site
13	2016, 06	Huangnan, Qinghai	Production site
14	2016, 06	Changdu, Tibet	Production site
15	2016, 06	Changdu, Tibet	Production site
16	2016, 06	Biru, Tibet	Production site
17	2016, 06	Suo, Tibet	Production site
18	2016, 06	Maqu, Gansu	Production site
19	2016, 06	Shiping, Yunnan	Production site
20	2016, 06	Ganzi, Sichuan	Production site
21	2016, 05	Litang, Sichuan	Production site
22	2016, 06	Gannan, Qinghai	Production site
23	2016, 06	Shannan, Tibet	Production site
24	2016, 06	Naqu, Tibet	Production site
25	2016, 06	Guoluo, Qinghai	Market
26	2016, 06	Guoluo, Qinghai	Market
27	2016, 06	Guoluo, Qinghai	Market
28	2016, 06	Biru, Tibet	Market
29	2016, 06	Maqu, Gansu	Market
30	2016, 06	Ganzi, Sichuan	Market
31	2016, 06	Yushu, Qinghai	Market
32	2016, 06	Guoluo, Qinghai	Pharmacy
33	2016, 06	Yushu, Qinghai	Pharmacy
34	2016, 06	Ganzi, Sichuan	Pharmacy

### Analysis of total arsenic in *C. sinensis*

#### Sample preparation

For total arsenic analysis, all samples were digested following the method officially listed in Pharmacopoeia of the People’s Republic of China (PPRC) [2]. Briefly, the tested samples were ground into powder and 0.25 g was placed into microwave digestion vessels (CEM Corporation, Matthews, NC, USA). An additional 8.0 mL HNO_3_ was mixed with the samples in the vessel. The microwave digestion procedures are shown in Table 2. After digestion, the samples were cooled to 60.0 °C, and the acid in the vessels was dispelled for 40.0 min. The digestion solution was then transferred into 50.0-mL volumetric flasks and diluted with Milli-Q water to volume.

**Table 2:** Microwave digestion program

	Step		
	I	II	III
Power (%)	100	Parameter	100
Time (min)	3	3	12
Temperature (°C)	120	150	200

#### Inductively coupled plasma mass spectrometry (ICP-MS) analysis of concentrations of total arsenic in *C. sinensis*

ICP-MS data were obtained using the Agilent 7700X ICP-MS (Agilent Technologies, Folsom, CA, USA). The ICP-MS system was calibrated before each batch of analysis using five point calibration curves (R^2^ > 0.999). ICP-MS measurements were executed under the following conditions: plasma gas flow rate: 15.0 L/min; peristaltic pump: 0.2 r/s; atomizing chamber temperature: 2.0 °C; auxiliary gas flow rate: 0.8 L/min; He gas flow rate: 5.0 mL/min; carrier gas flow rate: 0.8 L/min; RF power: 1550 W; 100 sweeps; three replicates; sampling depth: 10.0 mm.

For quality control, blanks and duplicate samples were analyzed during the procedure. Germanium (m/z 74) was used as the internal standard and added to the blanks, samples, and calibration standards to compensate for matrix effects and signal drift. The mean percent recovery of the internal standard solution was 102.6%; the mean percent recovery of total arsenic was 116.3% (n = 34).

### Concentration levels of arsenic speciation

#### Preparation of mixed arsenic reference standard solution

The mixed arsenic standard solution of six speciations comprising DMA, MMA, AsIII, AsV, AsB, and AsC was diluted to a series concentrations of 10.0, 20.0, 50.0, 100.0, 250.0, and 500.0 μg/L by Milli-Q water that was prepared for speciation analysis.

#### Sample preparation

The samples were ground into powder and 0.25 g was placed into the 50.0-mL glass tubes that were matched with the microwave extraction system (CEM Corporation, Matthews, NC, USA), after which 10.0 mL HNO_3_ (1.4 mol/L) were added to the glass tubes. The samples were extracted using the microwave extraction system at 70.0 °C for 10.0 min. After cooling to room temperature, the samples were filtrated through 0.22-μm membranes.

#### High performance liquid chromatography (HPLC)–ICP-MS analysis of the concentration levels of different speciation of arsenic in *C. sinensis*

The different speciation of arsenic was analyzed by HPLC–ICP-MS. HPLC conditions included a gradient HPLC system using (NH_4_)_2_CO_3_ (mobile phase A) and water (mobile phase B) at a flow rate of 0.5 mL/min to separate the analytes on an anion-exchange column (Agilent AS7 column, 4.6 × 250 mm, 5.0 μm particle size). The analysis was conducted according to the following procedures: for eluent A: 10.0% initial proportion; a linear increase to 50.0% at 3.0 min and to 100.0% at 4.0 min; keep to 11.0 min; linear decrease to 10.0% at 13.0 min; keep equilibrium for 17.0 min. The total acquisition time was 17.0 min. The injection volume was 10.0 μL.

### Health risk assessment of arsenic in *C. sinensis*

#### HI

Hazard index is calculated using the estimated weekly intake (EWI) for a contaminant divided by the provisional tolerable weekly intake (PTWI) [24]. The World Health Organization (WHO) recommended the PTWI for inorganic arsenic to be 15.0 μg/kg body weight (bw) [25]. If HI > 1.0, the risk is unacceptable [26].

$$ {\text{EWI}} = \;7 \times {\text{maximum}}\,{\text{daily}}\;{\text{consumption}}\,{\text{of}}\,C.\;sinensis \times {\text{concentration}}\,{\text{of}}\,{\text{inorganic}}\,{\text{arsenic}}/{\text{W}}. $$

According to PPRC, the recommended maximum daily consumption of *C. sinensis* is 9.0 g [2]. The concentration of inorganic arsenic is the average inorganic arsenic concentration detected; W is the average body weight. According to statistical data from the National Health and Family Planning Commission of the People’s Republic of China, the average adult female body weight and average adult male body weight in China in 2015 were 57.3 and 66.2 kg, respectively.

#### CR

Lifetime cancer risk for inorganic arsenic was expressed by the cancer slope factor (CSF) according to the U.S. Environmental Protection Agency (USEPA). If CR risk is larger than the acceptable lifetime risk of 10^−5^ considered by USEPA, there is a probability of a > 1 in 100,000 chance of an individual developing cancer [27]. CR is obtained by the following equation [28]:

$$ {\text{CR}} = {\text{EF}} \times {\text{Ed}} \times {\text{FIR}} \times {\text{C}} \times {\text{CFS}} \times 0.00 1/{\text{W}} \times {\text{AT}}, $$

where EF is the exposure frequency or number of exposure events per year (from 365.0 day/year for people who consumes *C. sinensis* every month to 30.0 day/year for people who consumes *C. sinensis* in only 1.0 month/year); Ed is the exposure duration and is equivalent to the average lifetime. According to the statistical data from WHO, the average lifetime of an adult Chinese female is 77.0 years and the average lifetime of adult Chinese male is 74.0 years; FIR is the *C. sinensis* ingestion rate. In this study, we used PPRC’s provision for *C. sinensis*, the maximum daily ingestion rate of which is 9.0 g/day [2]. CSF is the cancer slope factor for inorganic arsenic provided by USEPA, which is 1.5 (μg/g/day)^−1^. C is the arsenic concentration in *C. sinensis*; W is the average body weight; AT is the average exposure time for *C. sinensis* (365.0 day/year × Ed).

#### THQ

Target hazard quotient is calculated using the formula provided by USEPA as follows:

$$ {\text{THQ}} = {\text{EF}} \times {\text{Ed}} \times {\text{FIR}} \times {\text{C}} \times 0.001/{\text{RfD}} \times {\text{W}} \times {\text{AT}}, $$

where RfD is the oral reference dose for arsenic recommended as 3.0 × 10^−4^ μg/g/day. The other parameters in the equation are the same as those for CR. THQ is calculated using the ratio between the exposure and the reference dose. If THQ is < 1, considered by USEPA, no adverse health effects are expected as a result of exposure. If THQ is > 1, meaning that it is higher than the reference dose, the health of the exposed population is of concern [29].

#### Statistical analysis

A frequency distribution was performed to determine the relationship between the sum contents of As III and As V in *C. sinensis* and their frequencies using SPSS 19.0 (IBM Corporation, Armonk, NY, USA). Statistical analysis was performed to compute the significant differences among the sum contents of As III and As V in the endosclerotium, stroma, and the whole *C. sinensis* by one-way analysis of variance (ANOVA) using GraphPad 5.0 Software (San Diego, CA, USA), applying p < 0.001 as the minimum level of significance.

## Results

From the results of “Method validation” section in the present study, it was demonstrated that the four organic arsenic speciations (AsB, DMA, MMA, AsC) were not transformed to inorganic arsenic speciations or other organic arsenic compounds. But it is still not clear that except the four organic speciations, whether other organic arsenic speciations with unknown chemical structure, toxicity or risk, which were called “unknown risk arsenic compounds” in the present study, transformed to inorganic arsenic speciations or not. Nevertheless, considering the primary purpose of the study was to evaluate the risk of *C. sinensis* complying with the rule of risk maximization in order to protect most people. Therefore, in the process of health risk assessment, the risks of *C. sinensis* were maximized, and the unknown risk arsenic compounds were assumed as the inorganic arsenic with high toxicity to obtain a relatively conservative conclusion to protect most people. And the AsIII and AsV in the “Results” section in the study represents “AsIII and unknown risk arsenic compounds” and “AsV and unknown risk arsenic compounds”, respectively.

### Results of total arsenic and arsenic speciation analysis in *C. sinensis*

**Fig. 1:**
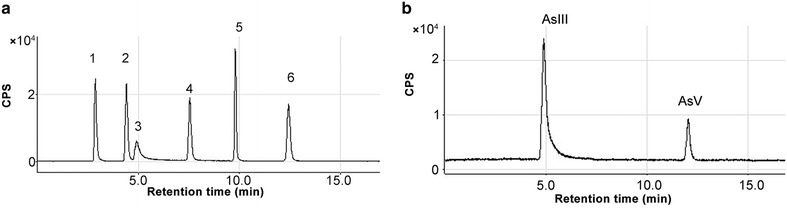
Representative ion chromatograms for arsenic speciation. **a** Arsenic speciation of mixed arsenic standard solution. 1–6 represented AsB, DMA, AsIII, MMA, AsC, and AsV, respectively. **b** Arsenic speciation in *Cordyceps sinensis*

The contents of arsenic speciation in 34 batches of *C. sinensis* were analyzed using the established HPLC–ICP-MS method. The representative ion chromatograms for typical separation of different arsenics are shown in Fig. 1. The contents of different arsenic speciation in the samples are shown in Table 3.

**Table 3:** Contents of different arsenic speciation in *Cordyceps sinensis* (mg/kg, n = 34)

AsB	DMA	AsIII^a^	AsC	MMA	AsV	Total arsenic
ND	ND	6.68 ± 2.68	ND	ND	1.84 ± 1.15	8.53 ± 3.49

*ND* not detected

^a^Data are represented as the mean ± SD

**Fig. 2:**
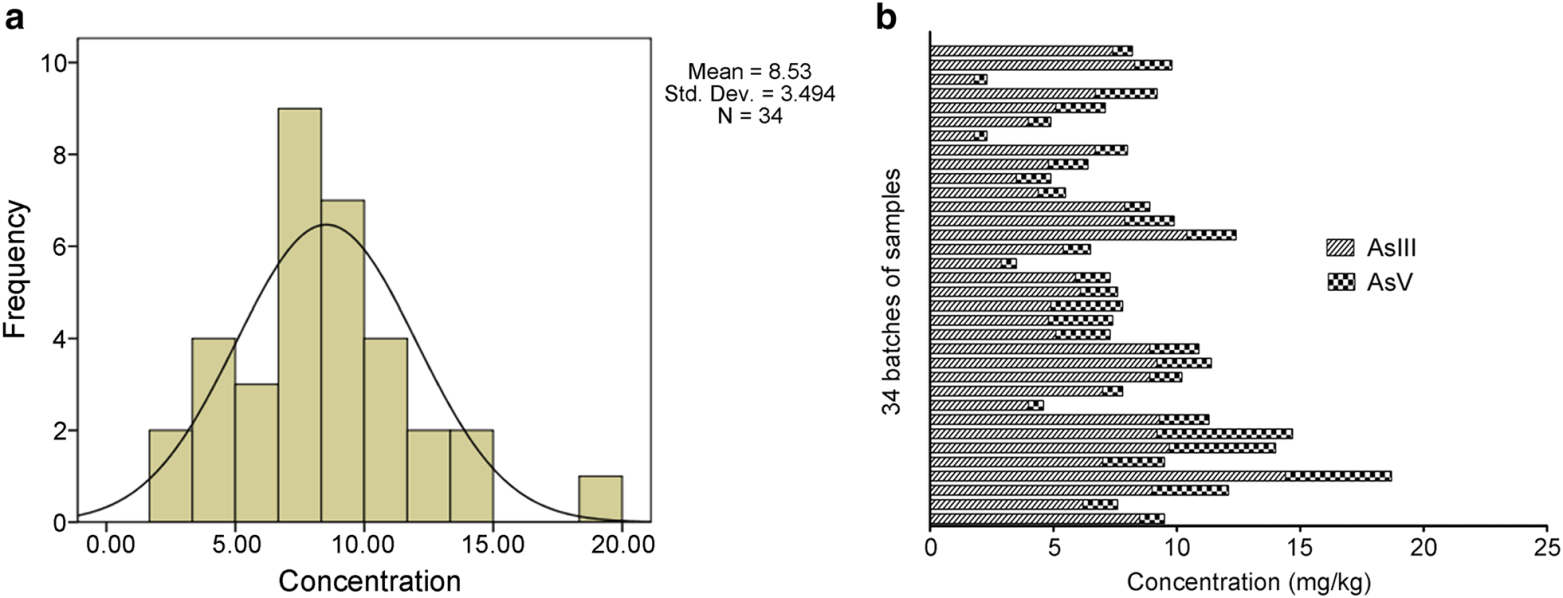
Statistical analysis of content of AsIII and AsV in 34 batches of samples. **a** Frequency distribution of the sum content of AsIII and AsV in samples. **b** Sum content of AsIII and AsV in samples

The analysis results for arsenic speciation (Fig. 1a) showed that different arsenic speciation was well separated. Figure 1b and Table 3 indicate that the dominant speciations in the *C. sinensis* samples were not four organic arsenic (AsB, DMA, AsC, and MMA) but As III and As V. Statistical analysis showed that the frequency distribution of the sum content of As III and As V in 34 batches of samples was normal, and the sum content of As III and As V in most samples (> 50.0%) ranged from 6.50 to 10.50 mg/kg (Fig. 2a). Furthermore, the total arsenic contents in 34 batches of *C. sinensis* were experimentally compared with the different arsenic speciation in the samples. The results showed that the total arsenic contents in *C. sinensis* were within the range of 2.12–15.51 mg/kg; the mean content was 8.32 mg/kg. The sum content of As III and As V in 34 batches of samples ranged from 2.31 to 18.71 mg/kg; the mean content was 8.53 mg/kg (Fig. 2b). The average deviation between the total arsenic contents and the sum contents of As III and As V was ~ 0.15%. We found that the extraction efficiency of the optimized procedure was > 99.0%, and the results from the ICP-MS method for the detection of total arsenic contents were in excellent agreement with the results obtained from the HPLC–ICP-MS method for the detection of all of the different speciations (Table 4).

**Table 4:** Extraction efficiency using the optimized method

AsIII (mg/kg)	AsV (mg/kg)	AsIII + AsV (mg/kg)^a^	Total arsenic (mg/kg)^b^	Extraction efficiency (%)^c^
6.67 ± 2.68^d^	1.84 ± 1.15	8.53 ± 3.49	8.32 ± 3.25	99.85 ± 0.007

^a^Sum of AsIII and AsV was determined by high-performance liquid chromatography coupled with inductively coupled plasma mass spectrometer spectrometry (HPLC–ICP-MS)

^b^Total arsenic was determined using ICP-MS after digestion

^c^Ratio of AsIII + AsV to total arsenic

^d^Data are represented as mean value ± standard deviation (n = 34)

**Fig. 3:**
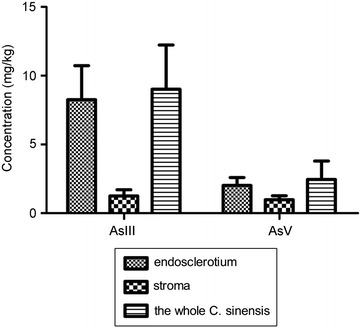
AsIII and AsV contents analyzed in fungal endosclerotium, stroma, and the whole *Cordyceps sinensis*

Moreover, arsenic speciation in fungal endosclerotium, stroma, and the whole *C. sinensis* of the five batches were analyzed. Statistical analysis indicated that the sum level of As III and As V in fungal endosclerotium was significantly higher (p < 0.001) than that in stroma. But there was no significant difference between the sum level of As III and As V in fungal endosclerotium and in the whole *C. sinensis* (p > 0.05). A clear trend was observed that both As III and As V were found mainly in the fungal endosclerotium (Table 5, Fig. 3).

**Table 5:** Arsenic speciation analysis in *Cordyceps sinensis* from different parts (mg/kg, n = 5)

Part	Concentration						
	AsB	DMA	AsIII^a^	AsC	MMA	AsV	Total
Endosclerotium	ND	ND	8.26 ± 2.47	ND	ND	2.02 ± 0.57	10.26 ± 2.79
Stroma	ND	ND	1.26 ± 0.45	ND	ND	0.98 ± 0.29	2.24 ± 0.48
The whole *C. sinensis*	ND	ND	9.02 ± 3.21	ND	ND	2.46 ± 1.33	11.54 ± 4.33

*ND* detected

^a^Data are represented as the mean ± SD

### Health risk assessment of arsenic in *C. sinensis*

Estimated weekly intake was calculated and compared to PTWI suggested by the Food and Agriculture Organization of the United Nations (FAO)/WHO expert to determine HI of females and males. HI of females and males for inorganic arsenic was 0.6 and 0.5, respectively, which was lower than the critical value of 1; therefore, as seen from HI values, the health risk associated with inorganic arsenic in *C. sinensis* was assumed to be acceptable.

To assess the cancer risk, CR vales were above the acceptable lifetime risk of 10^−5^ for all levels of assumed exposure for *C. sinensis* (Table 6). This indicated that even at the minimum stage of exposure, which was presumed to be 1.0 month/year, the probability of a > 1 in 100,000 chance of an individual developing cancer.

**Table 6:** THQ and CR estimated for inorganic arsenic at different levels of exposure

Level of exposure (months per year)	THQ		CR	
	Female	Male	Female	Male
1	0.36	0.32	1.6 × 10^−4^	1.4 × 10^−4^
2	0.73	0.63	3.3 × 10^−4^	2.8 × 10^−4^
6	2.19	1.89	9.9 × 10^−4^	8.4 × 10^−4^
12	4.38	3.78	2.0 × 10^−3^	1.7 × 10^−3^

Target hazard quotient proposed by USEPA is a complicated parameter used to estimate the potential health risks associated with long-term exposure to chemical pollutants by comparing the ingested amount of a contaminant with a standard reference dose. The inorganic arsenic THQ values (Table 6) were estimated to be < 1 for *C. sinensis* if the exposure was assumed to be < 2.0 months/year, especially for males; therefore, at exposure levels < 2.0 months/year, it is not likely to cause any deleterious effects to human health during a lifetime. On the contrary, for exposure levels > 3.0 months/year, the systemic effects of arsenic in *C. sinensis* were assumed to be of concern in terms of the health risks for both females and males; therefore, the maximum allowable *C. sinensis* rate for both males and females was stated as 2.0 months/year, which was the suggested THQ value for exposure levels to avoid the harmful systemic effects.

The THQ index used in risk assessment of arsenic offers a better picture than a simple parameter, such as the contents of arsenic in *C. sinensis*. It involves not only the content level of contaminants but also important information of exposure frequency, exposure duration, RfD, and body weight. The method for estimating THQ cannot be translated into a quantitative estimate on the probability of health effects occurring in a given exposed population, and it is unlikely to be proportional to the risk. THQ > 1 does not necessarily suggest that there will be definite adverse effects, but it does indicate that the systemic effects are assumed to be of concern [30, 31]. In our study, because of the differences in body weight, the THQ values were higher for females than males [32–34]. It should not be ignored that factors such as contaminant bioavailability, the uncertainty factor regarding RfD, and contaminant transfer rates from raw herbal medicines to decoction also play important roles in the THQ value [35]; therefore, THQ is designed to be a conservative estimate, and further research is needed to be able to offer guidance on applicable value choices for the risk assessment of harmful contaminants in traditional herbal medicines, including *C. sinensis.*

## Discussion

### Optimization of sample preparation for arsenic speciation analysis

The analysis of arsenic speciation in *C. sinensis* is challenging. An acceptable method should extract most of the arsenic from the matrix without altering speciation or producing toxic waste. For determining optimal extraction conditions, a variety of extraction methods have been developed. The extraction method (shaking, enzymolysis, ultrasonic extraction, and microwave extraction), extraction solvents (50.0% methanol; 50.0% acetonitrile; hydrochloric acid of 1.0, 5.0, and 10.0%; 1.4 mol/L HNO_3_; artificial gastric juice; and artificial intestinal juice), extraction temperatures (room temperature, 40.0, 50.0, or 70.0 °C) and extraction times (10.0, 20.0, or 120.0 min) were optimized. One of the criterions for selecting extraction method was based on the recovery, for which the degradation of the target analytes was expected to be minimum. The results demonstrated that microwave extraction was the most effective of all extraction methods. Extraction time was another factor that microwave extraction was applied in the present study. It was indicated that the target samples could be extracted completely within 10.0 min. As compared with other methods of extraction, microwave extraction took much less time. And a closed system of microwave extraction guaranteed a sealed experimental environment which prevented contamination [36].

Microwave extraction technology is a new and efficient extraction technology for the extraction of chemicals from food or herbal medicines. A high-frequency electromagnetic wave is necessary to penetrate the medium for extraction and then the heat energy is converted [36]. The cells rupture and the intracellular active ingredients discharge and dissolve in the extraction medium at a relatively low temperature. With these mechanisms, the extraction rate increases several times at a relatively low temperature and guarantees the quality of extraction to the highest extent. It was also observed that 1.4 mol/L HNO_3_ was the most efficient of all the solvents. Above all, the sample solutions were prepared by microwave extraction with 1.4 mol/L HNO_3_ for 10.0 min.

### Optimization of chromatographic condition

A variety of mobile phase systems ((NH_4_)_2_CO_3_–water, NH_4_HCO_3_–water, (NH_4_)_2_HPO_4_–water, and NH_4_H_2_PO_4_–water) were investigated in order to achieve an appropriate chromatographic behavior. The (NH_4_)_2_CO_3_–water system was selected because it offered the best peak shape and resolution of the target analytes. Different concentrations of (NH4)_2_CO_3_ solution and flow rate were also tested. Finally, 0.1 mol/L (NH_4_)_2_CO_3_–water eluting solvent system at a flow rate of 0.5 mL/min was preferred, because it produced a satisfactory separation efficiency, good ionization intensity and low baseline noise. Furthermore, the elution gradient programs were then optimized.

### Method validation

Linearity, limit of detection (LOD), limit of quantification (LOQ), precision, and the accuracy of the developed HPLC–ICP-MS method for the analysis of arsenic speciation were tested following the criteria for the validation of an analytical method. The precision of the method was tested using relative standard deviation (RSD).

The calibration curves for all the six speciations of arsenic were expressed by plotting the peak area (y) versus concentration (x) of each analyte, as shown in Table 7.

**Table 7:** Calibration curves and correlation coefficients of all arsenic speciations

Speciation	Calibration curves	r^2^
AsB	y = 1920x − 5817.1	0.9999
DMA	y = 1905x − 1457.1	1.0000
AsIII	y = 1070.7x − 5547	0.9993
AsC	y = 1743.2x − 1003.9	0.9999
MMA	y = 1833.5x + 7428.9	0.9988
AsV	y = 1844.8x + 882.21	0.9998

The samples with known contents were diluted to a series of concentrations and prepared and analyzed using the method previously described. LODs and LOQs based on the signal–noise ratios of three and ten times, was 2.0 and 10.0 ng/mL, respectively.

To confirm the repeatability of the developed method, six samples from the same source were extracted and analyzed using the previously mentioned method. The RSD value for inorganic arsenic and unknown risk arsenic compounds, the main speciation found in the target samples, was 3.90%, indicating good repeatability of the method.

The accuracy was determined by the mean recovery rate of the target samples. A known amount of the mixed standard solution was added to 0.25 g of the samples. The spiked samples were then extracted and analyzed using the method described above. As shown in Table 8, the mean recovery rates for different speciations of arsenic were in the range of 94.14–106.53%, with RSD values < 3.50%.

**Table 8:** Recovery rates of the developed method (n = 6)

Specifications	Original (ng)^a^	Spiked amount (ng)	Determined amount (ng)	Recovery rate (%)	RSD (%)
AsB	ND^b^	103.75	97.95 ± 2.38	94.41 ± 0.02	2.43
DMA	ND	99.50	106.00 ± 0.70	106.53 ± 0.007	0.66
AsIII	15.96 ± 0.01	100.20	110.29 ± 3.30	94.14 ± 0.03	3.49
MMA	ND	100.12	97.73 ± 2.26	97.61 ± 0.02	2.31
As III^c^	ND	100.02	108.88 ± 1.73	108.86 ± 0.01	1.59
As V^d^	3.39 ± 0.35	100.97	102.43 ± 1.22	98.09 ± 0.01	1.18

^a^Data are represented as the mean ± SD

^b^ND = not detected

^c^AsIII represents AsIII and unknown risk arsenic compounds

^d^AsV represents AsV and unknown risk arsenic compounds

## Conclusion

In this study, we developed the HPLC–ICP-MS method to determine the contents of arsenic speciation in *C. sinensis*. The optimized microwave extraction procedures, which were a new technology for pretreatment of traditional herbal medicines, saved time and resulted in a satisfactory extraction efficiency (> 99.0%). It was demonstrated that the main speciation of arsenic in *C. sinensis* were the inorganic arsenic and unknown risk arsenic compounds. We first assessed the health risk using HI, CR, and THQ parameters. HI suggested that the health risk for consumption of *C. sinensis* was acceptable. CR provided that even at the lowest exposure stage of 1.0 month/year, the risk of cancer should not be ignored. THQ indicated that at an exposure level < 2.0 months/year, ingestion was not likely to harm human health during a lifetime; however, if the exposure was > 3.0 months/year, the systemic effects of inorganic arsenic in *C. sinensis* might be of concern. These results indicating the recommended *C. sinensis* consumption rate of 2.0 months/year provided important input on the exposure suggestion or consumption limits to minimize potential health risks.

For *C. sinensis,* arsenic exposure is a significant worldwide environmental and health concern. It was reported that there were a number of organic arsenic compounds, however, the speciation, toxicity or risk were unclear [37]. For further research, there is still a lot to explore in terms of these unknown risk arsenic compounds. I hope our study played the role of “cast a brick to attract jade” to analyze arsenic speciations in *C. sinensis*. Continuous monitoring of arsenic speciation levels and the evaluation of the risks in *C. sinensis* are necessary. Further research is also needed in the interest of public health, including studying the techniques able to reduce heavy metal translocation in traditional herbal medicines. In the meantime, new methods by which to artificially reproduce *C. sinensis* with low arsenic residues are desirable.

The risk assessment methods for traditional herbal medicines are still in the exploration stages. We have focused more on determining contaminant contents in traditional herbal medicines; however, the contents of the contaminants at a high level or even exceeding the legal limits do not always represent risks for human health. Therefore, after obtaining the levels of contaminants by using modern technology, the methods of risk assessment for harmful residues or contaminants in traditional herbal medicines will be a new trend and of great significance. Thus, we hope that our study plays a role in providing new ideas for quality control and evaluation of traditional herbal medicines by the rule of risk maximization. We also hope that the combination of modern instrument analysis and risk assessment methods for traditional herbal medicines can be promoted and developed to scientifically evaluate possible risks regarding human health hazards.

## Additional file

**Additional file 1.** Minimum Standards of Reporting Checklist.

## Abbreviations

AsB: arsenobetaine
AsC: arsenocholine
CR: lifetime cancer risk
CSF: cancer slope factor
DMA: dimethyl arsenic
EWI: estimated weekly intake
HI: hazard index
HPLC–ICP-MS: high-performance liquid chromatography coupled with inductively coupled plasma mass spectrometry
ICP-MS: inductively coupled plasma mass spectrometry
LOD: limit of detection
LOQ: limit of quantification
MMA: monomethyl arsenic
PPRC: Pharmacopoeia of the People’s Republic of China
PTWI: provisional tolerable weekly intake
RSD: relative standard deviation
THQ: target hazard quotient
